# Innovations in paper-based analytical devices and portable absorption photometers for onsite analysis

**DOI:** 10.1007/s44211-025-00764-2

**Published:** 2025-04-18

**Authors:** Sasikarn Seetasang, Mika I. Umeda, Jianchao Ren, Takashi Kaneta

**Affiliations:** 1 Department of Chemistry, Faculty of Science and Technology, Thammasat University, Pathum Thani, 12120 Thailand; 2 National Institute of Technology, Yonago College, 4448 Hikona-Cho, Yonago, Tottori 683-8502 Japan; 3 Department of Chemistry, Okayama University, 3-1-1 Tsushimanaka, Okayama, 700-8530 Japan

**Keywords:** Point-of-care testing, Onsite analysis, Paper-based analytical device, Paired emitter–detector light-emitting diode, Photometer, Environmental analysis, Food analysis

## Abstract

Two types of analytical instruments and devices—one sophisticated high-performance instrument and another portable device—have been the focus of recent trends in analytical science. The necessity of point-of-care testing and onsite analysis has accelerated the advancement of high-performance, user-friendly portable analytical devices such as paper-based analytical devices (PADs) and light-emitting diode-based portable photometers. In this review, we summarize our achievements in the study of PADs and portable photometers. Several types of PADs are capable of performing titrations, metal ion analysis, and food analysis, while photometers, which consist of paired emitter–detector light-emitting diode (PEDD) photometers, are used for thiocyanate and herbicide analysis. These PADs and photometers permit the onsite determination of real environmental, body fluid, and food samples when an equipped laboratory is unavailable.

**Graphical Abstract:**

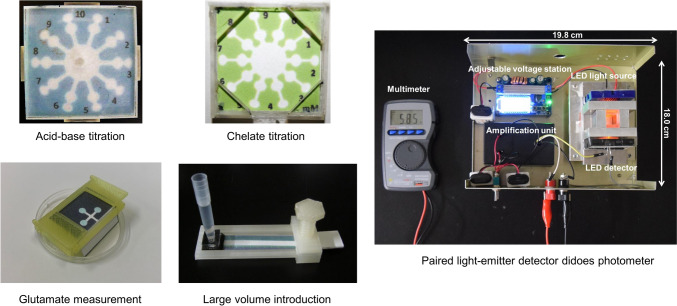

## Introduction

Advanced technologies rely on sophisticated analytical instruments based on spectrometry, electrochemistry, and separation methods including chromatography and electrophoresis. The analytical instruments establish selective and sensitive analyses in the medical, pharmaceutical, agricultural, food, and environmental sciences. Thus, analytical science plays a crucial role in research across all areas of chemistry and has contributed significantly to scientific progress.

Researchers have also developed numerous portable devices for onsite measurements in the field, which includes medical diagnostics, food quality control, and environmental analysis. Portable absorbance detectors have facilitated colorimetric analysis outside of equipped laboratories and are now commercially available. Researchers have also reported different types of miniaturized and portable absorbance detectors with light-emitting diodes and photo detectors [1–6]. On the other hand, paper-based analytical devices (PADs) have served as an effective technology for point-of-care testing and onsite analysis since 2007 [7]. Many review articles have summarized the general concepts and applications [8–10] such as use in medical diagnostics and pharmaceutics [11, 12], onsite environmental analysis [13], and the home-based testing of food quality [14]. In addition to our own work, herein we also review other studies of PADs and the miniaturized detectors that have been developed to permit onsite analysis [15].

These portable analytical devices are considered to meet the ASSURED criteria proposed by the World Health Organization (WHO) and are anticipated to play a significant role in achieving the United Nations' Sustainable Development Goals (SDGs) because some of them are strongly related to chemical analyses. For example, water quality analysis is essential for supplying safe water. Currently, 2 billion people in the world have no access to safe drinking water, and 3.6 billion people use water that is untreated. The WHO reports that safe water, sanitation and hygiene (WASH) could prevent 1.4 million deaths per year [16]. Water pollution remains a major issue in many regions, which include developing countries, and is being addressed as one of the SDGs. For continuous water quality management in areas with insufficient facilities and power supply, it is essential to achieve low-cost and simple chemical analyses onsite.

Over the past decade, we have developed various PADs and portable absorbance detectors to facilitate chemical measurements in the field. In this review, we summarize our work on the development and application of PADs and portable absorbance detectors employing light-emitting diodes (LEDs) as both light sources and detectors. Our research includes the development of PADs based on novel principles and their application to the determination of target analytes in real-world samples. Additionally, we have developed portable absorbance photometers for the analyses of biological samples and environmental monitoring. Several types of PADs with different designs and detection principles have been developed for the measurements of acids, bases, metal ions, and small molecules. Conversely, portable photometers have been constructed using the principle of paired emitter–detector light-emitting diodes (PEDDs) [3–6] because LEDs have several advantages for optical measurements: small size, low-power operation, and the ability to function as both a light source and a light detector. These characteristics of LEDs have established the miniaturization of several optical devices. We have developed PEDD-based photometers that are suitable for onsite analysis because of their portability. In this review, we describe the PADs and PEDD-based photometers and their applications to real samples rather than spiked ones.

## Fabrication of PADs

Many researchers have reported several methods to fabricate PADs. In one of the first reports, PADs were fabricated by photolithography [7], and thereafter researchers developed several fabrication methods such as wax printing, ink-jet printing, cutting, stamping, and hand-drawn methods [17]. Among them, photolithography, wax printing, ink-jet-printing, and cutting methods permit flexible, precise, and reproducible designs. Conversely, wax printing [18], ink-jet printing [19], and cutting [20, 21] methods are facile and cost-effective. Unfortunately, the manufacturers have stopped supplying wax printers, although such a process is the most convenient to fabricate PADs. Therefore, a simple, easy, low-cost, and precise fabrication method is needed for the development of PADs.

Many materials have been employed to fabricate hydrophobic barriers on paper substrates. Our group fabricates PADs using a wax printer provided by Xerox that is not commercially available at the present time. We also use a cutting method via a cutting plotter that is one of the easier methods for fabrication. Future research requires a simple fabrication method that will be low-cost, easily available, and precise, and could serve as an alternative to wax printing.

## PADs for titrations

Although classic titration requires a wide range of glassware, large volumes of samples and standard solutions, and troublesome operations, these methods confer several advantages. The most important and useful characteristic of titration methods is the lack of a need for calibration curves. Thus, the results directly lead to the amount or concentration of an analyte without the need to construct a calibration curve. To obtain these advantages when using PADs, we developed titration PADs that permit the measurement of acids and bases [22] as well as calcium (Ca^2+^) and magnesium (Mg^2+^) [23].

**Fig. 1:**
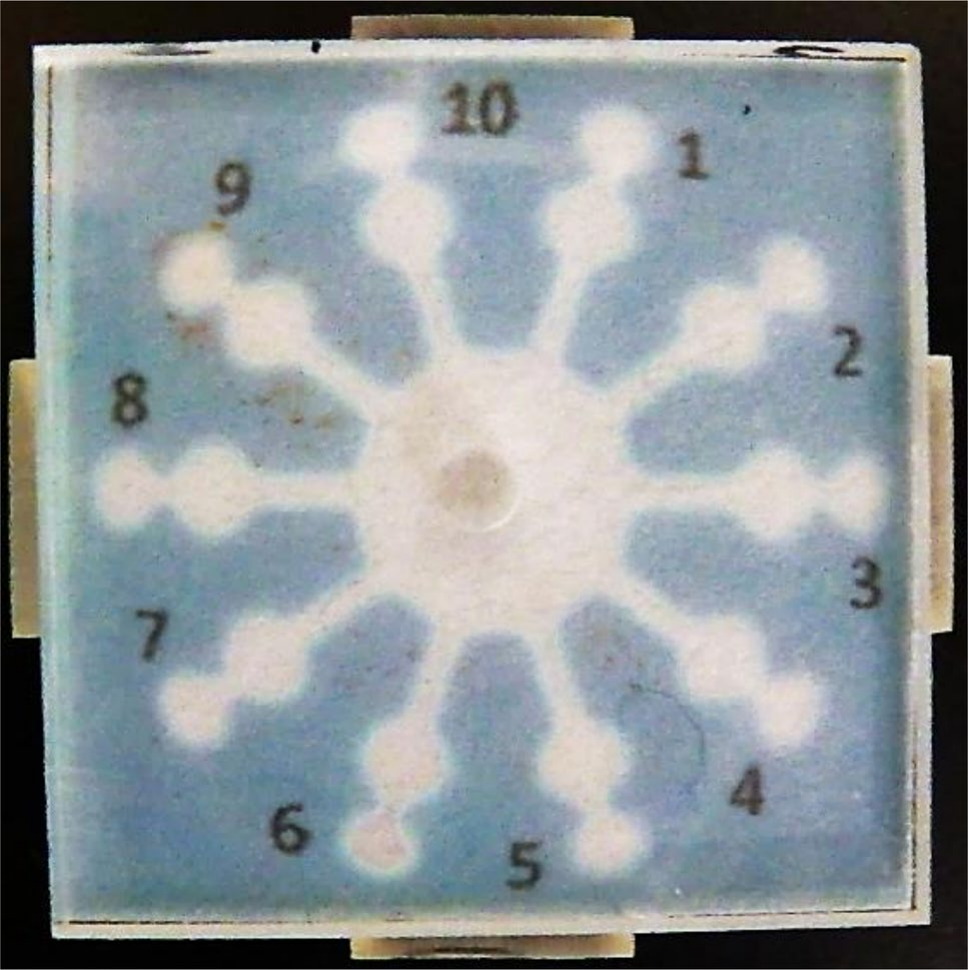
PAD for acid–base titration (adapted from Ref. [22] with permission from The American Chemical Society)

The PAD for acid–base titrations was designed as shown in Fig. 1 [22]. This PAD consists of a circle connected with ten radially located channels. Each channel equips two circle zones for reaction and detection. When a solution is dropped into the introduction zone (center circle), it spreads into a circular shape due to capillary action, while simultaneously flowing into the ten channels. Titrant solutions with different concentrations are pre-deposited in each reaction zone, and an indicator is introduced into a detection zone. After dropping the sample solution into the sample introduction zone, the results are obtained in less than a minute. The titrant (primary standard substance) is pre-deposited into each reaction zone where it reacts with the introduced sample solution, and excess analyte that is not consumed by the titrant reaches the detection zone, which causes a color change. Therefore, the boundary concentration at which a color change occurs in the detection zone is the end point, and the result can be read visually.

Acid–base titration has been used to quantify known concentrations of strong acids and strong bases such as hydrochloric acid, nitric acid, and sodium hydroxide with an endpoint that involves the use of phenolphthalein as an indicator to correctly identify the analyte concentrations. PADs have been used to accurately measure the concentration of acetic acid, which is a weak acid. However, ammonia showed an unclear endpoint due to the deviation of the equivalent point from the color-change range of phenolphthalein. This problem was solved by changing the indicator from phenolphthalein to bromocresol purple. Therefore, the PADs for acid–base titrations could also be used to measure weak acids and weak bases when a suitable indicator is selected.

To demonstrate the applicability of the titration PADs to onsite analysis, we brought the titration PADs to a volcano where acidic hot spring water flows out, and we measured the acidity of the hot spring water outside. The hot spring water samples reacted immediately when contacting the PADs for measurement. Then, samples were brought to the laboratory and a classic titration method was performed the next day. The results of the PADs showed good agreement with those of the classic acid–base titration performed in the laboratory.

The PADs also achieved chelation titrations of Ca^2+^ and Mg^2+^ with ethylenediaminetetraacetic acid (EDTA) as the titrant [23], while using the same basic detection principle as that of classic chelate titration. The PADs were prepared by adding a buffer solution at pH 10 or 13 to all hydrophilic zones and depositing EDTA solutions with different concentrations in the reaction zones with an indicator at a constant concentration in the detection zones. Metal ions in a sample solution react with EDTA in the reaction zones, and subsequently flow into the detection zones. When the amount of the metal ions exceeds the amount of EDTA in a reaction zone, the excess metal ions react with the indicator, which results in a color change from blue (free indicator) to purple (complex of the indicator and the metal ion). In the PADs for chelate titration, the length of the flow channel connecting the reaction zones and the detection zones had to be longer than in the case of acid–base titration because of the time required for EDTA and buffer components to dissolve in water. By lengthening the flow channel, we were able to control the time that the solution flows from reaction zones to detection zones. The concentrations of Ca^2+^ and Mg^2+^ were determined by measuring the total amount of Ca^2+^ and Mg^2+^ at pH 10, and then subtracting the separately measured Ca^2+^ concentration at pH 13 from the total amount to calculate the Mg^2+^ concentration.

In general, an ammonia buffer solution adjusts the pH at 10 for measuring the total amount, and a KOH buffer solution plays the role of a buffer at pH 13 to measure only Ca^2+^ in classic chelate titrations. In PADs, however, the reagents added to the channels and zones are in a dried state, so volatile ammonia is not suitable as a buffer. Therefore, instead of using ammonia as a buffer, we used N-cyclohexyl-3-aminopropanesulfonic acid (CAPS), which is a solid at room temperature and has a pKa of 10.6.

The PADs were used to determine the total concentrations of Ca^2+^ and Mg^2+^ (pH 10) and the concentration of Ca^2+^ (pH 13) at the minimum measurable concentrations of 1.0 and 4.0 mM, respectively. The measurable minimum concentration of Ca^2+^ was higher than that of the total concentration due to the adsorption of Ca^2+^ by the paper substrate at pH 13. In practical applications, the PADs were used to successfully measure mixtures of Ca^2+^ and Mg^2+^ and were also applied to the analyses of commercially available mineral water, seawater, and river water. These results demonstrated that the PADs could be applied to the differential quantification of Ca^2+^ and Mg^2+^. The results of the PADs were validated via classic titration and showed consistent results.

## PADs for heavy metal ions

The measurement of heavy metal ions using PADs was first reported by Nie et al. using electrochemical detection [24] and by Hossain et al. using colorimetric detection [25]. Henry’s group has reported the measurement of metal ions using PADs based on color intensity [26] and color distance [27]. We have also reported PADs for measuring heavy metal ions based on colorimetry, colorimetric distance measurement, and chemiluminescence methods. The development of PADs has permitted onsite measurements of iron(II), nickel(II), copper(II), and chromium(III) and (VI).

### Measurement of iron(II)

Popular colorimetric reagents for iron(II) (Fe(II)) include 1, 10-phenanthroline (Phen) and bathophenanthroline (Bphen), which are also excellent reagents for the measurement of Fe(II) using PADs. We developed PADs for measuring Fe(III) from the color intensity of the colorimetric reagent, Phen. The principle is the same as that reported by Henry’s group [26] where hydroxylamine, polyacrylic acid, and a Phen solution were dissolved in an acetate buffer solution and added to the four detection zones arranged around the center circle [28]. When a solution containing Fe(II) and Fe(III) was dropped into the sample introduction zone, an Fe(II)–Phen complex was formed in the detection zone, which turned the color of the solution to red. The intensity of the red color could be used to quantify the concentration of iron ions by using software to process the image. When these PADs were applied to the determination of different forms of iron ions in hot spring water, the results were consistent with those obtained by spectrophotometry. The limit of detection (LOD) of these PADs was estimated to be 12 ppm.

To further improve the sensitivity of iron measurement, we equipped a PAD with a distance readout [29]. This PAD had a sample reservoir that would allow the continuous flow of a sample in the detection channel. A 1-mL micropipette tip served as a sample reservoir that was fixed with a PDMS block on the introduction zone. The detection channel contained Bphen as a colorimetric reagent, and Fe(II) ions reacted with Bphen as they flowed into the detection channel, which resulted in a colored bar the length of which depended on the concentration and volume of the Fe(II) solution. Thus, 1 mL of a sample solution was continuously introduced into the PAD with a distance readout, and the LOD was significantly improved when it was lowered to 20 ppb, which is comparable to the sensitivity of inductively coupled plasma-optical emission spectroscopy. The LOD was 150-fold lower than that of the previously reported PAD with a distance readout using a small sample volume (50 μL). The PADs were also successfully applied to the measurement of iron ions in hot spring-water with a concentration that had a range of tens of ppb.

### Measurement of chromium

**Fig. 2:**
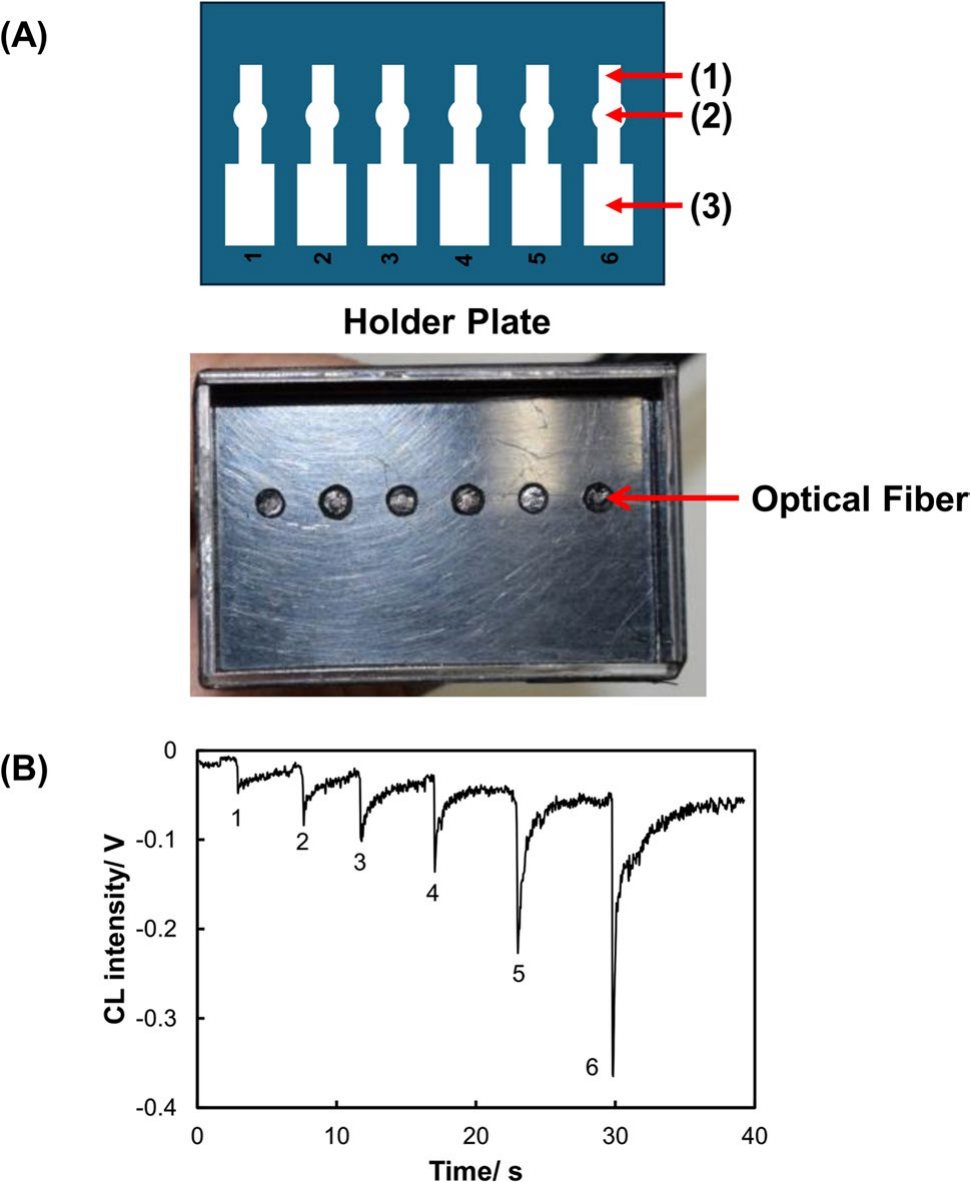
Design of a PAD and holder for chemiluminescence detection of Cr(III). **A** PAD and holder; (1) reagent introduction zone, (2) sample introduction zone, (3) waste channel, **B** response curve for Cr(III) (adapted from Ref. [30])

We have developed a compact chemiluminescence detector for PADs to improve the sensitivity for Cr(III) [30]. The PADs were equipped with six flow channels—each possessing an injection zone, a reaction zone, and a waste zone (Fig. 2). The chemiluminescence detector for the PADs was constructed using optical fibers. The ends of six optical fibers were located in the reaction zones of the six channels on the PADs. The optical fibers were bundled in a manner that placed each at the front of the photovoltaic surface of a small photomultiplier tube module. The reactions are based on catalytic enhancement in the chemiluminescence of luminol when in the presence of Cr(III). Six Cr(III) solutions were dropped into the reaction zones in advance and dried, and subsequently the chemiluminescent reagents were added to the injection zone to generate chemiluminescence in the reaction zones. The chemiluminescence signals quantified Cr(III) with a LOD of 0.020 ppm and a limit of quantification of 0.066 ppm.

**Fig. 3:**
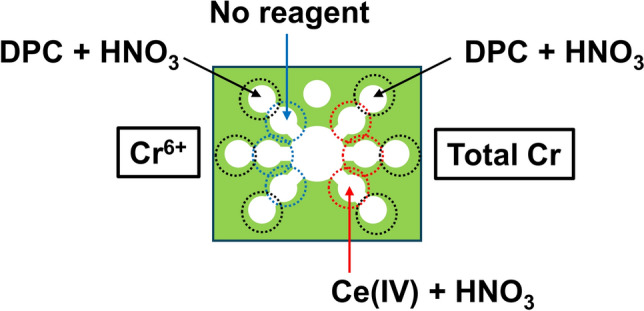
Design of a PAD for the speciation of Cr(III) and Cr(VI) (adapted from Ref. [31] with permission from Springer Nature)

Although we accomplished a sensitive measurement of Cr(III) via chemiluminescence, Cr(IV) should also be a target in environmental science because of its high toxicity. A differential determination between these two compounds is an important issue because the chromium in Cr(III) and Cr(VI) in aqueous solutions normally exists in different states of oxidation. Therefore, we developed PADs with the capability of discriminating between Cr(III) and Cr(VI). A colorimetric reaction is based on the complex formation of Cr(VI) with diphenylcarbazide (DPC) and on the oxidation of Cr(III) to Cr(VI) via Ce(IV) [31]. The design of the PADs is shown in Fig. 3. The sample reservoir is located in the center of the PADs, and three flow channels extend radially from the central circle to the left and right sides, respectively. The left channels detect Cr(VI) while the right channels detect the total amount of Cr(III) and Cr(VI). The left channels contain only DPC in the detection zones whereas the right channels consist of the reaction zones for the oxidation with Ce(IV) and the detection zones for the complex formation with DPC. In the right channels, Cr(III) was converted to Cr(VI) by Ce(IV) in the reaction zones located before the detection zones. Although the conversion efficiency was almost 100%, we noticed that Ce(IV) inhibited the complex formation reaction between DPC and Cr(VI). As a consequence, we achieved a speciation of chromium by correcting the effect of Ce(IV), which allowed measurement of the total chromium concentration.

On the other hand, the utility of the colorimetric PADs using DPC was confirmed during a remote analysis of Cr(VI) in Ethiopia. The PADs permitted measurement of the total chromium concentration in soils and crops in remote industrial areas of Ethiopia. The PADs were designed and fabricated in Japan, and then transported to Ethiopia. The results of that study revealed that the soil and crops in industrialized areas of Ethiopia were significantly contaminated as extremely high concentrations of chromium were quantified by the PADs, which demonstrated the potential to achieve remote analysis of environmental pollutants [32].

### Coupling of coprecipitation preconcentration for Cu(II), Ni(II), and Cr(VI)

PADs with colorimetry are the most frequently investigated type, because the processing of data is simple and easy. However, the colorimetric PADs generally show sensitivity that is lower than that of standard spectrophotometric methods. To enhance sensitivity, the PADs were successfully improved by coupling with coprecipitation for preconcentrating Cu(II) [33], Ni(II) [34], and Cr(VI) [35]. In the coprecipitation preconcentration of these metal ions, Al(OH)_3_ was an excellent precipitant. The coprecipitation method concentrated each metal ion, and this resulted in an improvement that was 100–250-fold greater than the existing limit of detection. The resultant PADs showed a level of sensitivity that agreed well with that of inductively coupled plasma-optical emission spectroscopy. The proposed methods made it possible to analyze trace levels of metal ions in environmental water with high sensitivity. Thus, the coupling of coprecipitation enrichment with the PADs is expected to be useful for environmental monitoring without the need for expensive analytical equipment, particularly when used in developing countries.

## PADs for inorganic anions

The determination of inorganic anions is also a challenge for environmental analysis. For example, nitrogen and phosphor are important nutrients that also could be contaminants at high concentration levels. Thus, for nitrites and nitrates we developed PADs that have a layered structure [36]. The detection zones contain a Griess reagent that generates a red color product when reacting with nitrite. The red color appears when nitrites are introduced from the sample introduction zone via a micropipette. Images of the detection zones were captured by a scanner and processed to obtain the color intensity. A calibration curve was constructed by plotting the color intensity against the concentration of nitrites.

The PADs for nitrites are also employed for the determination of nitrates via a coupling that reduces nitrate to nitrite. This reduction is achieved by sandwiching layers of zinc powder between the introduction and detection zones. When the reaction zone layers contain zinc, the total concentration of nitrites and nitrates is expected to be measured, and the nitrate concentration is then calculated by subtracting the nitrite concentration from the total concentration of nitrites and nitrates. However, we noticed that an incomplete reduction of nitrate to nitrite decreased the color intensity of nitrite in the presence of zinc. Therefore, when determining both nitrites and nitrates, we proposed the construction of three calibration curves for nitrites in the absence of zinc, nitrates in the presence of zinc, and nitrites in the presence of zinc. Using the layered PADs in the presence and the absence of a reaction zone between the introduction and detection zones, the measurements of nitrites and nitrates could be completed within 1 h.

The measurement of phosphate is also important, because it is an important nutrient for the growth of plants and animals whereas high concentration levels contaminate natural water. The analysis of phosphate is generally achieved via spectrophotometry using the molybdenum blue method. The principle of the molybdenum blue method is also applicable to the PADs, as shown in previous studies [37, 38]. Those studies, however, have reported difficulties in the use of onsite analysis. For example, the reagents must be added to begin the measurement [38] and samples must be precisely introduced via micropipette. To improve these issues, we employed a dipping method for sample introduction that requires no micropipette; a holder is fabricated using a 3D printer to fix the PAD, and a homemade photo studio box is used to capture the image of the PAD onsite.

Among the previously reported PADs for phosphate, a layered PAD would be the most promising version because it prevents the reaction of a molybdate with a reductant [37]. However, the previously reported PAD requires a precise volume of sample introduction with a micropipette. Conversely, the proposed dipping method is user-friendly because there is no need to use a micropipette to introduce a sample. The sample is introduced by immersing the introduction zone into the sample solution at a certain time, and the introduction volume is controlled according to time.

Therefore, we developed a PAD for phosphate determination, which employs a layered structure and a dipping method for sample introduction to achieve portable and user-friendly analysis [39]. In the layered PAD for the measurement of phosphate, the first layer contains a molybdenum reagent consisting of ammonium molybdate, sulfuric acid, and potassium antimonyl tartrate. A second layer consists of ascorbic acid as a reductant. Then, the first and second layers must firmly contact one another to efficiently produce the blue product. To that end, a holder to fix the two layers was fabricated via a 3D printer. In addition, the images of the devices were captured with a smartphone using a homemade photo studio box to maintain constant illumination conditions. Using these simple tools allows the measurement of phosphates outside of laboratories or in poorly equipped laboratories.

While the PAD measured phosphate at the concentration range of 1–10 ppm, further low concentrations, e.g. lower than 1 ppm, must be accurately quantified for monitoring contamination of natural water. For practical analysis, we succeeded in quantifying the measurement of phosphate concentrations by combining solid-phase extraction with anion-exchange resins. The solid-phase extraction improved the limit of detection by a factor of 10, which achieved a sensitivity sufficient to measure phosphate in real samples including river water, soil, and toothpaste.

Many PADs determine the concentration of an analyte by measuring the color intensity generated by a reaction product that is read using an external device such as a digital camera, scanner, personal computer, and smartphone. By contrast, a distance readout visually provides the signal and quantifies an analyte without the use of external devices, as demonstrated in the measurement of Fe(II) in our study [29]. We proposed a method to determine borate using a distance readout in which the length of the colored channel depends on the retention behavior of brilliant green dye, which has a positive charge, according to the principle of ion-pair chromatography. Initially, borate reacts with chromotropic acid to form a negatively charged complex, and then the complex produces an ion-pair in the brilliant green dye. Brilliant green dye was printed at the entrance of the detection channel for a distance readout, and subsequently the complex of borate with chromotropic acid was introduced into the detection channel. The brilliant green dye was dissolved in the solution containing the complex of borate with chromotropic acid and flowed in a retaining manner in the detection channel based on the chromatographic principle. We oxidized the paper substrate to introduce carboxyl groups with negative charges. Therefore, the brilliant green dye with a positive charge strongly interacts with the negatively charged surface of paper while the ion-pair of the complex and the brilliant green dye weakly interact with the negatively charged surface of paper. Consequently, the length of the color of the brilliant green dye depended on the concentration of the borate complex combined with chromotropic acid [40].

The colored distance of brilliant green dye is visually readable, so the borate concentration in the sample solution is determined by measuring the distance by simply using a ruler. The PADs were printed with a 107 mm straight channel extending from the circular sample introduction zone, and a band of brilliant green dye was applied to the connection between them. After the sample was introduced, the retardation factor (R_f_) was calculated from the distance to the top of the flowing solution and the distance of the color band. Since R_f_ correlated with the borate concentration, we could quantify the borate concentration using the calibration curve. We applied the PADs to the measurement of the borate concentration using a commercially available eye dropper. The obtained values were comparable to those measured using the conventional azomethine-H method.

### PADs for food analysis

The screening of chemical contaminants, biological hazards, and allergens in food is a critical concern for the food industry, as these substances can cause serious illness and, more importantly, could lead to the death of consumers. From the suppliers' perspective, this issue incurs significant expense and causes delays in delivery to customers when products are recalled. Currently, many conventional methods such as chromatography and spectroscopy [41–44] are employed for food quality control and to deliver accurate analytical results. However, these methods require well-equipped laboratories, skilled professionals, expensive and large quantities of reagents, and involve complex sample preparation before analysis, all of which are necessary to achieve reliable results. Therefore, these high-performance methods are not always ideal for onsite food surveillance.

PADs show promising potential to revolutionize food safety and quality control by meeting the ASSURED criteria suggested by the WHO [45]: affordable, sensitive, specific, user-friendly, rapid, robust, equipment-free, and deliverable [46]. Consequently, the development of PADs for applications in the food industry has been proposed over the past few decades. The developed PADs cover a wide range of chemical contaminants, biological hazards, and allergenic compounds such as pesticides [47, 48], inorganic ions [49, 50], organic compounds [51, 52], pathogens [53, 54], heavy metals [55, 56], and allergens [57, 58]. Several approaches have been explored to bring PAD features in line with ASSURED criteria. However, most PADs continue to require micropipettes for precise and accurate sample introduction, which poses a challenge for user-friendly design and home-use applications. Consequently, many researchers have attempted to develop PADs that function without the need for micropipettes.

**Fig. 4:**
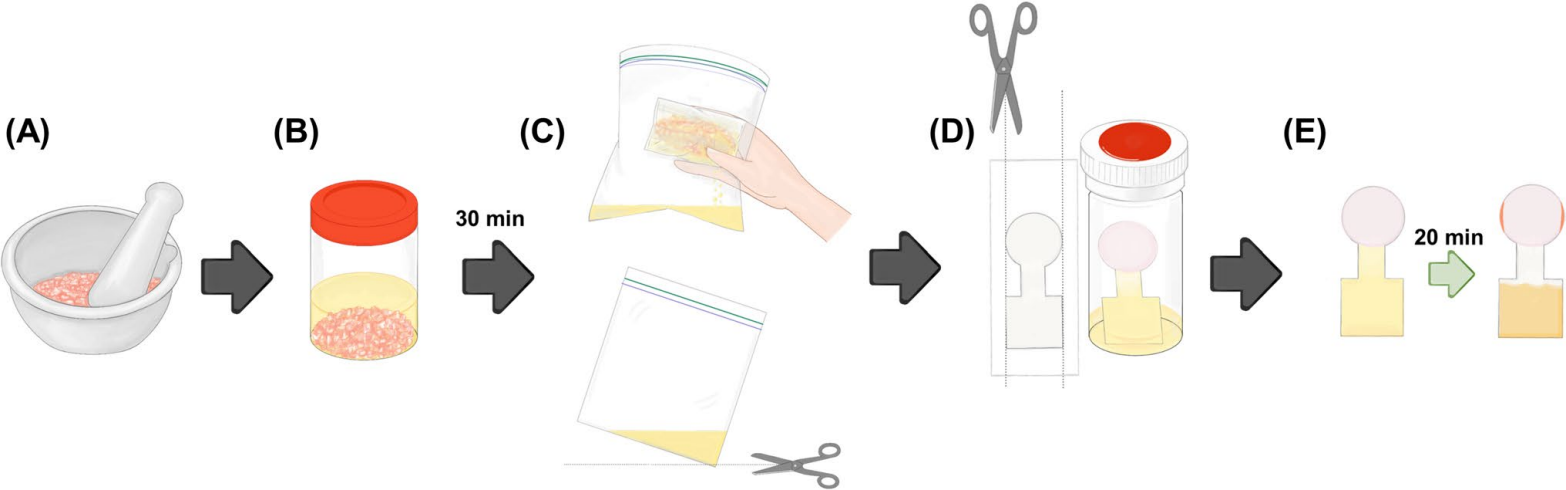
Experimental procedures for the analysis of indole in shrimp: **A** shrimp sample was prepared by grinding; **B** the ground shrimp was extracted by EtOAc; **C** shrimp meat and EtOAc solution were filtered; (D) the PAD was cut to create holes (green arrows) before immersing the PAD into the vial to allow a colorimetric reaction; and, (E) the PAD was removed from the vial before performing data analysis. The orange arrows indicate the accumulation of astaxanthin at the solvent exit (adapted from Ref. [59] with permission from The American Chemical Society)

In 2022, Seetasang and Kaneta introduced dip-and-read PADs equipped with chromatographic separation for indole analysis in shrimp [59]. The design of PADs consists of a sample introduction zone, a flow channel, and a detection zone. After depositing Ehrlich’s reagent in the detection zone to react with the indole compound, the zone was laminated using a heat press to prevent solvent evaporation during sample introduction via the dipping method. Since the reaction occurred in a 100% organic solvent, it was essential to perform it in a closed-bottom environment. The PADs demonstrated versatility, as the results were independent of both sample volume and dipping angle. To verify this, the authors fixed the indole concentration and varied the analyte’s volumes. The PADs were dipped into the vials having different volumes without adjusting the dipping angles. Results from 25 PADs (five replications at each volume) showed no significant differences in color intensity, with a %RSD of less than 5%. The versatility of the PADs was further validated by six untrained volunteers with little-to-no experience in chemistry experiments. They were provided only with instructions on measuring indole concentrations at different levels. Five volunteers correctly estimated the indole concentration as prepared. One volunteer, however, reported an incorrect concentration of 5 ppm due to the similarity of the colors at 2.5 ppm in the provided color chart, which indicated the need for further improvement to avoid misestimation. These results confirmed that the developed PADs are user-friendly and suitable for home-use applications by untrained individuals. Finally, the dip-and-read PADs were used for indole analysis in shrimp to monitor shrimp degradation. Figure 4 illustrates the steps of the analysis: (A) sample preparation, (B) extraction, (C) filtration, (D) PAD preparation, and (E) analysis. The results obtained using the developed methodology were compared with those from the standard high-performance liquid chromatography (HPLC) method, and no significant differences were found between the two methods, which indicates that the developed methodology is applicable for real shrimp sample analysis.

**Fig. 5:**
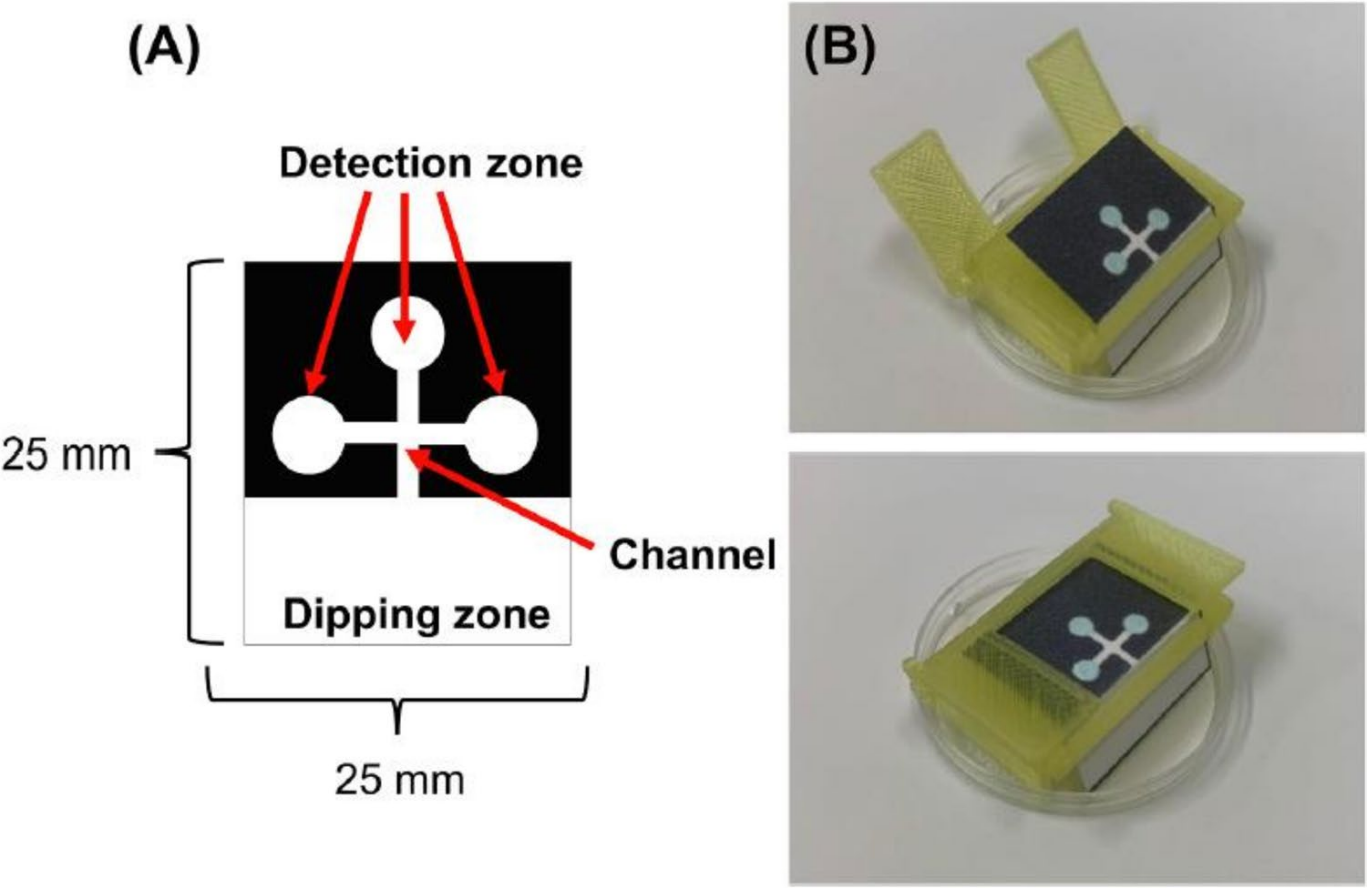
Designs of the PAD and the 3D-holder fabricated using a 3D printer: **A** the designed PAD for glutamate determination; and, **B** the 3D-holder with the PAD—A sample is added to the tray placed at the bottom of the 3D-holder (adapted from Ref. [60] with permission from Elsevier)

Danchana et al. investigated the presence of glutamate in food samples using an enzymatic reaction to produce a deep blue-colored product [60]. The proposed PADs included a sample introduction zone connected to a channel that directed the sample solution to three detection zones (Fig. 5A). These PADs also eliminated the need for pipettes by being perpendicularly immersed into the sample solution and bent at right angles using a 3D-printed holder that enabled simultaneous flow into the three detection zones (Fig. 5B). However, there was an issue with inhomogeneous color distribution when introducing the solution. To address this, an anionic poly(acrylic acid) was mixed with the reagents and applied to the detection zones. This improved the color homogeneity because the anionic polymer retains cationic methylene blue and forms stable ion pairs. Finally, the developed methodology was applied to analyze glutamate in soup powder, cookies, sauce, and juice. The results obtained from the developed method were compared with those using a commercial Glutamate Assay Kit-WST for microtiter plate assay. The calculated absolute t value (1.95) was lower than the critical t value (2.57) for all samples at 95% confidence, which suggests that the PADs are applicable to glutamate analysis in various matrices of food samples.

## Summary of PADs

The developed PADs are summarized in Table 1, which provides details on analytes, reagents, detection methods, measurable ranges, and limits of detection (LODs). We have previously reported various types of PADs designed to enhance sensitivity, portability, and user-friendliness. These PADs have enabled the analysis of acids, bases, various metal ions, and nutrients in environmental samples, as well as certain organic molecules in food samples. These studies highlight the potential utility of PADs for on-site analysis of environmental and food samples without the need for large, expensive instruments that require high levels of power consumption.

**Table 1:** Summary of the developed PADs

	Analyte	Reagent	Measurable range	Detection method	LOD	Refs.
PADs for titration	Acids and bases	Phenolphthalein (indicator)	5 × 10^–3^–1.0 M	Counting the number of zones with color change	5 mM	[22]
	Calcium and magnesium	BT and calcon (indicator)	0.5–100 mM (total of calcium and magnesium)		0.5 mM (total of calcium and magnesium)	[23]
			4–100 mM (calcium)		4 mM (calcium)	
PADs for heavy metal ions	Fe(II) and Fe(III)	1,10-Phenanthroline	40–350 ppm	Color intensity	12 ppm	[28]
		Bathophenanthroline	20–1000 ppb (nonlinear)	Distance (large volume introduction)	20 ppb	[29]
	Cr(III)	Luminol	0.05–100 ppm	Chemiluminescence	0.020 ppm	[30]
	Cr(III) and Cr(VI)	1,5-Diphenylcarbazide	0.1–60 mg L^−1^ (Cr(III))	Color intensity	0.08 mg L^−1^ (Cr(III))	[31]
			0.02–100 mg L^−1^ (Cr(VI))		0.008 mg L^−1^ (Cr(VI))	
PADs for heavy metal ions with coprecipitation concentration	Cu(II)	Bathocuproine	0.01–2.00 mg L^−1^	Color intensity (coprecipitation with Al(OH)_3_)	0.003 mg L^−1^	[33]
	Ni(II)	Dimethylglyoxime	0.03–2.00 mg L^−1^		0.009 mg L^−1^	[34]
	Cr(VI)	1,5-Diphenylcarbazide	0.005–2.000 mg L^−1^		0.0015 mg L^−1^	[35]
PADs for inorganic anions	Nitrite and nitrate	Griess reagent	0.05–1.5 mg L^−1^ (nitrite) 1–10 mg L^−1^ (nitrate)	Color intensity	0.1 mg L^−1^ (nitrite) 4.2 mg L^−1^ (nitrate)	[36]
	Phosphate	Molybdenum reagent	0.05–1 ppm	Color intensity (preconcentration with solid phase extraction)	0.089 ppm	[39]
	Borate	Chromotropic acid and brilliant green	1–3 mM	Distance	0.3 mM	[40]
PADs for food analysis	Indole	p-Dimethylaminobenzaldehyde	1.0–20.0 ppm	Color intensity	0.36 ppm	[59]
	Glutamate	Glutamate oxidase, horseradish peroxidase, and N-benzoyl leucomethylene blue	5 × 10^–6^–10^–2^ M	Color intensity	1 × 10^–6^ M	[60]

### Other challenges

User-friendliness is one of the issues to be addressed for achieving onsite analysis. In general, a micropipette must be employed for sample introduction because the introduction volume influences the signal intensity in many detection schemes. As shown in food analysis, whereas the dipping method permitted sample introduction without a micropipette, precise time control is essential to obtain reproducible results. Thus, we proposed a PAD with a distance readout that does not require a micropipette for the sample introduction. The PAD consists of a detection channel for distance readout and a storage channel that estimates the introduced volume [61].

The distance-based detection channel was connected to a storage channel that indicates the volume of a sample introduced into the PAD. An analyte in the sample solution reacts with a colorimetric reagent deposited into the distance-based detection channel, and the sample solution successively flows into the storage channel where the volume is measured. Therefore, the ratio of the lengths of the detection channel and that of the storage channel are constant for a sample containing a certain concentration even when different volumes are introduced into the PAD. Therefore, the PADs are user-friendly because they permit volume-independent quantification via the use of a dropper instead of a micropipette. The proposed PADs were applied to the determinations of iron and bovine serum albumin using bathophenanthroline and tetrabromophenol blue as colorimetric reagents, respectively. The calibration curves showed good linear relationships with coefficients of 0.989 for iron and 0.994 for bovine serum albumin when introducing the samples without precise control of the introduction volume.

Stability is also an important issue for onsite analysis because PADs must be stored and transported to a sample site to measure the analyte. The most important issue for improving the stability of the PAD is degradation of the reagents deposited on the PAD. Thus, we attempted to stabilize the hydrogen peroxide deposited on the PAD for the enzymatic reaction of horseradish peroxidase. Our finding indicated that poly(vinyl alcohol) (PVA) suppressed degradation of the hydrogen peroxide deposited on the paper [62].

Hydrogen peroxide and 3,3′,5,5′-tetramethylbenzidine were deposited as substrates, and horseradish peroxidase was added as the analyte. The presence of PVA significantly suppressed the degradation of hydrogen peroxide. The stability of hydrogen peroxide without PVA was degraded after one day under room temperature. However, storage of the PADs at 4 °C in a refrigerator extended the stability of the hydrogen peroxide containing 2% PVA by as much as 30 days.

Currently, compact devices and instruments are being developed and are now commercially available. For example, some miniaturized pH meters and conductometers operate only with a button battery and can be held in one hand. Thus, potentiometric titrations and conductometric titrations could be achieved onsite using these miniaturized devices. For this purpose, one of the issues is transportation of reagent solutions, particularly that of titrant solutions. Transportation of solutions is sometimes difficult because of possible leakage, degradation, and safety. Therefore, we proposed the use of pieces of paper as reagent containers.

Reagent-deposited pieces of paper were employed for conductometric titrations with a compact conductometer, potentiometric titration with a compact pH sensor, and a conventional spectrophotometer [63]. The pieces of paper were fabricated by wax printing to form a hydrophilic area that contained a constant amount of reagent. Surprisingly, the pieces of paper without the reagent increased the conductivity of water gradually due to the release of sodium salts, whereas the pH of NaOH was decreased because of the acidity of the functional groups in the paper. When sulfamic acid as an acid, Na_2_CO_3_ as a base, and BaCl_2_ as a metal salt were deposited on the pieces of paper, the reagents were released from the pieces of paper at different amounts. Sulfamic acid and Na_2_CO_3_ were released in quantities of 58 and 73% into water, whereas 100% of the BaCl_2_ was released. The conductometric titrations of NaOH, HCl, and Na_2_SO_4_, and the spectrophotometry of Fe^2+^ all were achieved using pieces of paper that contained sulfamic acid, Na_2_CO_3_, BaCl_2_, and 1,10-phenanthroline because the pieces of paper released the reagents quantitatively into the solution. Thus, the reagent-deposited pieces of paper are compatible for combining with miniaturized sensors and devices for onsite analysis.

### Portable absorption photometer with paired emitter–detector and light-emitting diodes

A photometer coupled with light-emitting diodes (LEDs) has been developed as a potential spectroscopic device. The unique properties of LEDs make them powerful optical components in miniaturized photometers. These properties include a long lifetime, a wide range of colors (from UV to IR), rapid response, high intensity, small size (millimeter scale), and safety at low-voltage operation [64, 65]. Also, the utilization of LEDs confers further advantages such as low-cost for setup and straightforward operation. Therefore, a pair of LEDs has been employed as the optical part of a miniaturized photometer. Over the past few decades, researchers such as D. Diamond’s group have used LEDs in spectroscopic devices as both a light source and a light detector that has prompted the concept of a paired emitter–detector light-emitting diode (PEDD)-based photometric device, for which a mathematical model was developed to relate their time-based strategy to the Beer–Lambert law [6, 66]. L. Tymecki and co-workers developed simple photometers using the PEDD setup and built LegoTM blocks as a cuvette holder for pH measurement [4, 67]. The use of LEDs as both light sources and detectors not only provides the previously mentioned advantages but also simplifies the analysis of quantitative results. This is due to the fact that when an LED acts as a light detector, it produces voltage proportional to the transmitted light after passing through a sample solution [67]. Additionally, using an LED as a light source enhances the selectivity of the photometer. This is because LEDs, when used as light detectors, are intrinsically bandwidth-limited, which reduces interference from other light bandwidths [68]. As a result of these significant advantages of using the PEDD setup in photometers, our group has proposed three generations of them.

### Miniaturized photometer with PEDDs

**Fig. 6:**
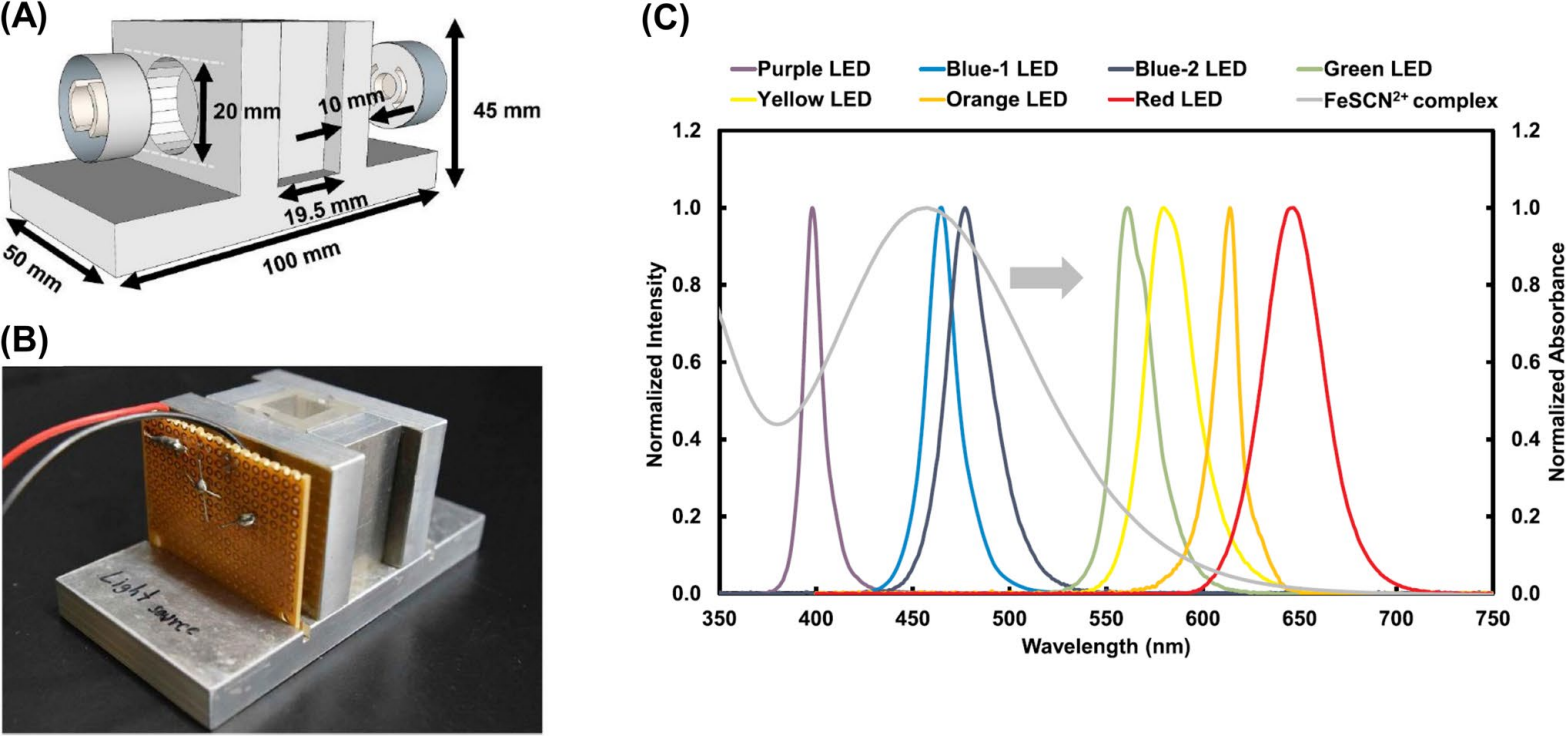
**A** The scheme; **B** photo of the PEDD-based photometer; and, **C** the absorption spectrum of the FeSCN^2+^ complex (gray line, *λ*_max_ = 457 nm) and emission spectra of several LEDs (adapted from Ref. [69] with permission from the Elsevier)

The construction of a photometer with PEDDs consists of three main tasks: (1) design of a cuvette holder, (2) selection of LEDs as a light source and light detector, and (3) design of an electric circuit. The cuvette holder was designed with a top rectangular channel that fits a 1-cm plastic cuvette and two-sided circular channels for plastic lenses to focus the dispersing light of the LED. The holder was constructed in-house using aluminum plates, as shown in Fig. 6A, B [69]. Next, the LED light source was selected based on an emission wavelength that matched the absorption wavelength of the colored complex with that of the analyte. Constant voltage was supplied to the LED light source and was controlled via the power supply. Finally, an amplification circuit consisting of an operational amplifier, rechargeable batteries, and resistors was used to enhance the photovoltaic power from the LED light detector. A multimeter operated in voltmeter mode was connected to the output of the amplification circuit. It is important to note that rechargeable batteries were suitable for obtaining reproducible results because decreases in battery power significantly affected the reproducibility of the output from the LED light detector.

The performance of the miniaturized PEDD-based photometer was evaluated using it to investigate thiocyanate (SCN^−^) in the saliva of non-smokers and smokers [69]. As previously mentioned, the selection of LEDs was based on the absorbance of an analyte. In this case, under acidic conditions, SCN^−^ forms a complex with ferric ion (Fe^3+^), which is referred to as the FeSCN^2+^ complex. Figure 6C displays the overlap of the spectra between the metal complex (λ_max_ at 457 nm) and the emitted light of several LEDs. Acomparison with other LEDs shows that the metal complex more effectively absorbed the emitted light from the blue-1 LED (λ_max_ at 465 nm). Therefore, the blue-1 LED was selected as a light source and light detector for SCN^−^ analysis. The results revealed that SCN^−^ concentrations in non-smokers were lower than 1 mmol L⁻^1^, while in smokers, they were approximately fivefold higher. This suggests that the detoxification of cyanide to SCN^−^ occurs more in smokers than in non-smokers due to their exposure to hydrogen cyanide gas during smoking. The results obtained by the present method agreed well with those from the solvent extraction method [70], which is considered as a standard protocol for SCN^−^ analysis. Therefore, this result proved that the developed photometer is reliable and applicable for real-world applications.

### Portable PEDD-based photometer operated by rechargeable batteries

To serve people in remote areas, where access to bulky instruments is difficult and there is a lack of professional users, the development of a miniaturized portable photometer should be prioritized. The fabrication was accomplished by utilizing rechargeable batteries as reported by several research groups. These studies cover a wide range of wavelengths that commonly span light from the UV–visible region [71–73] to the infrared region [74–76].

**Fig. 7:**
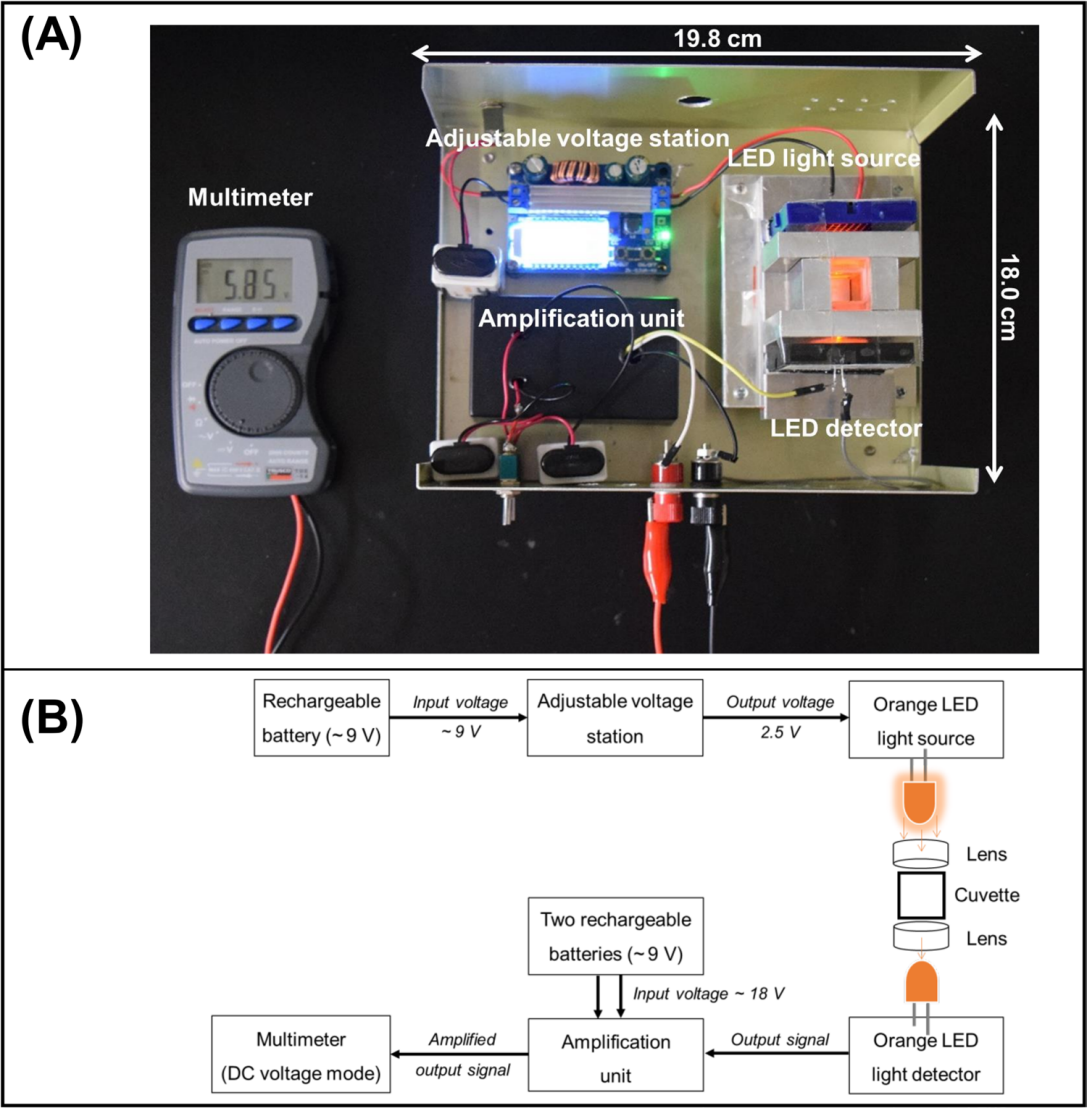
**A** Photograph and **B** schematic diagram of the portable PEDD detection device operated by rechargeable dry cell batteries connected with a multimeter (adapted from Ref. [77] with permission from the Elsevier)

A photograph and a schematic diagram of the portable photometer with PEDDs in the visible region appear in Fig. 7A, B, respectively. The entire system requires only three 9 V dry cell batteries for operation. One battery, connected to an adjustable voltage regulator, supplied a constant voltage to the LED light source. The other two batteries powered the amplification unit that interfaced with the LED light detector. The LEDs with plastic lenses (2.2 × 1.4 cm, 95% transmittance) were positioned on opposite sides of the cuvette chamber, and followed the standard layout of a spectrophotometer. The photovoltaic signal generated by the LED light detector was measured using a multimeter in DC voltage mode. The size of this developed photometer, housed in a black aluminum box, is approximately 18 × 20 cm, which makes it compact, portable, and suitable for portability and onsite analysis.

The miniaturized portable photometer was used for the onsite analysis of paraquat in the water of rice fields in Japan and Thailand [77]. Paraquat is a toxic chemical that is widely used as a non-selective herbicide to eliminate weeds and grasses in rice fields during the pre-sowing stage. Paraquat is highly toxic to humans, and has a lethal dose (LD_50_) that ranges from approximately 3–5 mg per kg of body weight. Even a small amount ingested orally could be fatal, as there is no antidote available [78]. This underscores the critical need to monitor paraquat residue in the environment that should enable timely warnings and assistance to farmers to prevent potential health hazards.

To analyze paraquat using colorimetric measurement by the developed photometer, the compound must undergo a redox reaction with sodium dithionite to produce a blue paraquat radical. However, sodium dithionite is unstable in the presence of oxygen in solution. Therefore, it is essential to ensure its stability during transportation to remote areas for onsite analysis. Additionally, an excess of the reducing agent is required to fully convert paraquat into a paraquat radical. Consequently, solid sodium dithionite could be added directly to the sample solution in any amount, as any unreacted dithionite does not affect the absorbance of the paraquat radical. Orange LEDs (λ_max_ at 609 nm) were employed in the portable photometer to measure the absorption of the paraquat radical (λ_max_ at 603 nm). The developed device was applied to analyze paraquat in water samples taken from rice fields. The analysis showed that there was no paraquat in any of the samples, which could have been due to strong adsorption between paraquat and soil particles and/or to complications with the sampling conditions. To demonstrate the absorption of paraquat by soil, an artificial rice field was constructed with a soil layer and a water layer. Paraquat was sprayed into the water layer, and its concentrations in both the water and soil were continuously monitored daily. The analysis showed that paraquat gradually decreased within three days in the water and could be found in the soil on the fourth day. These results were consistent with HPLC analysis, which emphasizes the applicability of this portable photometer for the onsite measurement of paraquat in water samples.

### Portable two-color PEDD-based photometer operated by rechargeable batteries

Multi-color detection is one of the challenges of using LEDs in photometric detection devices for measuring absorbance in the investigation of multiple analytes. For several years, researchers have reported using LEDs in detection systems. However, these systems typically require either complicated electrical circuits or an additional plug-in system to accomplish simultaneous analyses [79–81]. In 2021, our group reported the simple fabrication of a completely portable photometric device equipped with multi-color LEDs, which enabled the simultaneous analysis of multiple analytes.

**Fig. 8:**
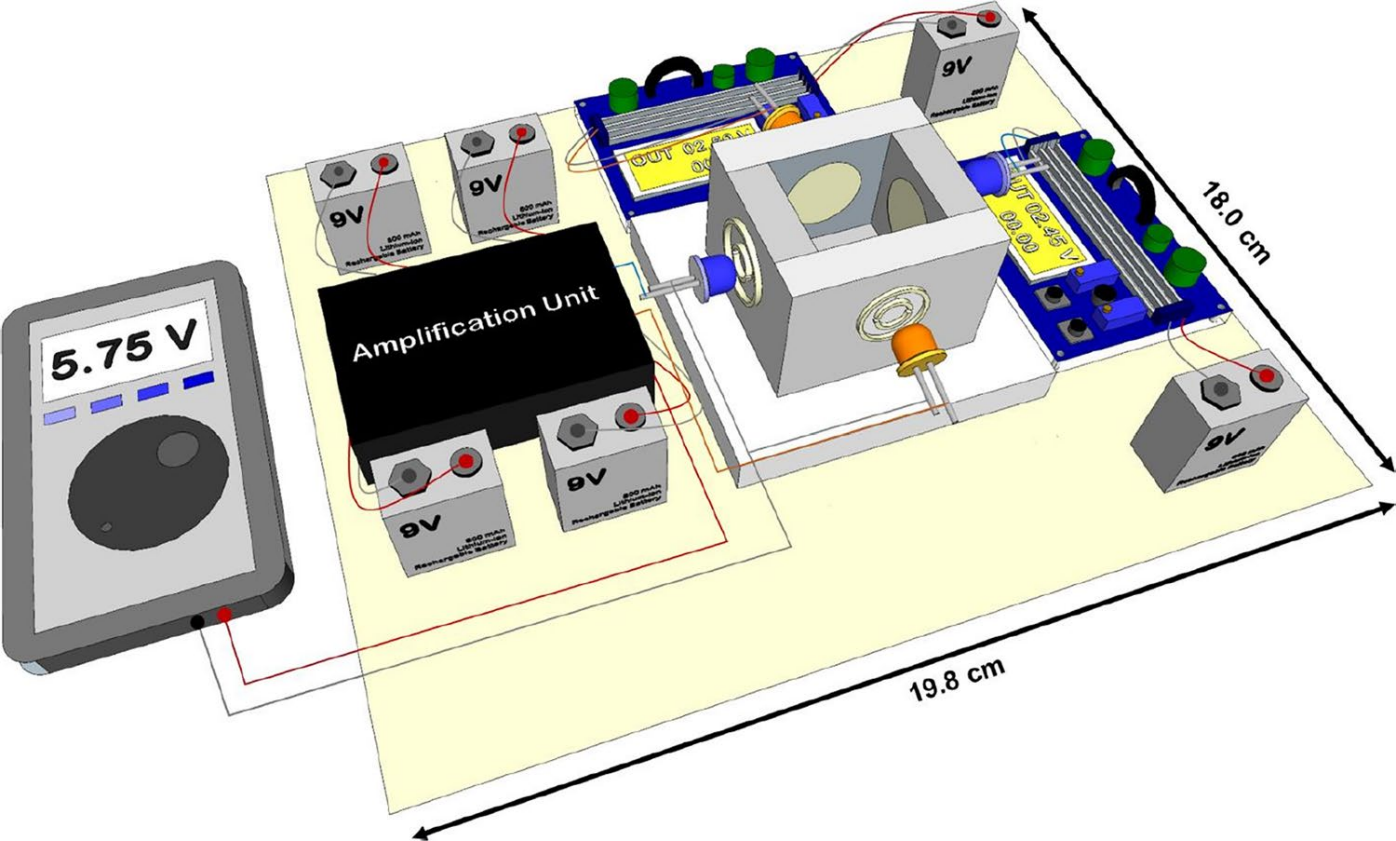
Three-dimensional drawing of the developed portable two-color photometer with paired light emitter–detector diodes for dual analysis of paraquat and diquat (adapted from Ref. [82] with permission from the Elsevier)

A new portable photometer equipped with a PEDD setup of two colors was developed [82]. This photometer was designed to overcome the interference issue between paraquat and diquat observed in previous work [77]. In this work, the photometer required two sets of power supply and amplification units for each color of the PEDD setup. Therefore, six batteries were required for the operation. Four sides of the cuvette holder were drilled in a circular pattern to accommodate the LEDs and lenses. Pairs of LEDs with the same color were oriented toward each other to function as both the light source and the light detector (Fig. 8). The cuvette holder also has a rectangular top channel for inserting the cuvette during analysis. All the components were placed in an aluminum box (18.0 × 19.8 × 9.0 cm), which made it suitable for portability and onsite applications. A multimeter was connected with the photometer system outside of the box and was assigned to display the photovoltaic signals generated by the two LEDs of different colors acting as light detectors. The developed photometer meets the ASSURED criteria (Affordable, Sensitive, Specific, User-friendly, Rapid and robust, Equipment-free, and Deliverable to end-users) as suggested by the WHO [46]. Notably, this setup proved to be both deliverable to end-users and affordable, with a total fabrication cost of approximately 85 USD.

Paraquat and diquat are toxic herbicides extensively used in agricultural countries. In 1985, the Japanese Association of Rural Medicine began promoting the co-formulation of these chemicals to prevent their intentional use in suicides or murders, as well as accidental exposure during agricultural activities, which predominantly occurred in rural areas of Japan [83]. Additionally, their similar chemical structures posed a challenge to accurate analysis because they could interfere with each other during chemical reactions. As a result, a portable two-color LED photometer was developed to analyze both toxic herbicides onsite. In this work, two redox reactions were used: (1) dithiothreitol-reduced diquat that generates a red-colored diquat radical (λ_max_ at 495 nm) and (2) sodium dithionite that reduces diquat and paraquat and produces a green-colored diquat radical (λ_max_ at 771 nm) and a blue-colored paraquat radical (λ_max_ at 603 nm), respectively. Based on the absorption characteristics of the targeted radical compounds, blue and orange LEDs were employed. The blue and orange LEDs have emission maximum wavelengths at 472 and 609 nm, respectively. The proposed methodology for multi-analyte analysis requires three calibration curves: two for diquat radicals (at 472 and 609 nm) and one for paraquat radicals (at 609 nm). The following steps were used to accurately determine the concentrations of diquat and paraquat from their mixture:

1. Measure the concentration of diquat radicals at 472 nm.
2. Convert the known concentration of diquat radicals into absorbance at 609 nm.
3. Subtract the calculated absorbance of diquat radicals from the absorbance of the mixture solution at 609 nm to isolate the absorbance of paraquat radicals.
4. Convert the absorbance of paraquat radicals to determine the paraquat concentration.

The reliability of this proposed method was demonstrated via analysis of a paraquat and diquat mixture in a commercial herbicide. The results were compared with those obtained using HPLC. This comparison confirmed that the proposed method accurately determines the concentrations of these herbicides, and no significant differences were noted between the two methods.

## Summary of PEDD-based photometers

Table 2 summarizes the developed PEDD-based photometers and highlights their features, applications, and analytical performance. The first-generation device enabled the analysis of thiocyanate ions in saliva, offering a low-cost solution despite requiring a plugged power supply. The second-generation device enhanced portability by operating solely on rechargeable dry cell batteries, and this facilitated the determination of paraquat in rice field soil. The third-generation device further improved analysis accuracy by addressing interference in paraquat detection through the use of two-color PEDDs. This two-color PEDD-based photometer allows the simultaneous determination of paraquat and diquat at different wavelengths without cross-interference.

**Table 2:** Summary of the developed PEDD-based photometers

	Power supply	Wavelengths of LEDs (nm)	Analyte	Measurable range	LOD	Refs.
First generation	Tabletop type	457	Thiocyanate ion	0.05–0.75 mM	0.01 mM	[69]
Second generation	Rechargeable dry cell battery (portable)	609	Paraquat	2.0–40.0 mg L^−1^	0.5 mg L^−1^	[77]
Third generation	Rechargeable dry cell battery (portable)	609 472	Paraquat Diquat	2.0–40.0 mg L^−1^ 2.5–40 mg L^−1^	0.56 mg L^−1^ 0.8 mg L^−1^	[82]

## Conclusions

Over the past two centuries, researchers have developed numerous sophisticated analytical instruments and continue to explore new versions to enhance their analytical capabilities. On the other hand, modern society demands portable and user-friendly analytical devices for chemical analysis in environmental, food, and medical fields to meet the SDGs, which heavily depend on onsite analysis and point-of-care testing. Therefore, numerous researchers are dedicating their efforts to the development of devices to satisfy the ASSURED criteria. In this review article, we described the PADs and miniaturized PEDD-based photometers developed by our group. We will continue our efforts to develop portable devices to enhance their analytical performance in terms of affordability, selectivity, sensitivity, precision, portability, and user-friendliness. We hope that our studies will provide valuable insights for future research in the areas of onsite analysis and point-of-care testing.

## Data Availability

The data used in the current study are available from the corresponding author upon reasonable request.

## References

[1] K. Danchana, P. Phansi, C.T. de Souza, S.L.C. Ferreira, V. Cerdà, Talanta **206**, 120250 (2020). 10.1016/j.talanta.2019.120250 31514846 10.1016/j.talanta.2019.120250

[2] K. Danchana, V. Cerdà, Talanta 216, 120977 (2020). 10.1016/j.talanta.2020.120977 10.1016/j.talanta.2020.120977 32456928

[3] M. O’Toole, K.T. Lau, B. Shazmann, R. Shepherd, P.N. Nesterenko, B. Paull, D. Diamond, Analyst **131**, 938 (2006). 10.1039/B602846B 17028728 10.1039/b602846b

[4] Ł Tymecki, M. Pokrzywnicka, R. Koncki, Analyst **133**, 1501 (2008). 10.1039/B807127F 18936826 10.1039/b807127f

[5] M. O’Toole, K.T. Lau, D. Diamond, Talanta **66**, 1340 (2005). 10.1016/j.talanta.2005.01.054 18970127 10.1016/j.talanta.2005.01.054

[6] K.T. Lau, S. Baldwin, M. O’Toole, R. Shepherd, W.J. Yerazunis, S. Izuo, S. Ueyama, D. Diamond, Anal. Chim. Acta **557**, 111 (2006). 10.1016/j.aca.2005.10.046

[7] A.W. Martinez, S.T. Phillips, M.J. Butte, G.M. Whitesides, Angew. Chem. **119**, 1340 (2007). 10.1002/ange.200603817

[8] D.M. Cate, J.A. Adkins, J. Mettakoonpitak, C.S. Henry, Anal. Chem. **87**, 19 (2015). 10.1021/ac503968p 25375292 10.1021/ac503968p

[9] Y. Yang, E. Noviana, M.P. Nguyen, B.J. Geiss, D.S. Dandy, C.S. Henry, Anal. Chem. **89**, 71 (2017). 10.1021/acs.analchem.6b04581 27936612 10.1021/acs.analchem.6b04581

[10] E. Noviana, T. Ozer, C.S. Carrell, J.S. Link, C. McMahon, I. Jang, C.S. Henry, Chem. Rev. **121**, 11835 (2021). 10.1021/acs.chemrev.0c01335 34125526 10.1021/acs.chemrev.0c01335

[11] E. Noviana, D.B. Carrão, R. Pratiwi, C.S. Henry, Anal. Chim. Acta **1116**, 70 (2020). 10.1016/j.aca.2020.03.013 32389191 10.1016/j.aca.2020.03.013

[12] N. Jiang, N.D. Tansukawat, L. Gonzalez-Macia, H.C. Ates, C. Dincer, F. Guder, S. Tasoglu, A.K. Yetisen, ACS Sens. **6**, 2108 (2021). 10.1021/acssensors.1c00669 34076428 10.1021/acssensors.1c00669

[13] N.A. Meredith, C. Quinn, D.M. Cate, T.H. Reilly, J. Volckens, C.S. Henry, Analyst **141**, 1874 (2016). 10.1039/C5AN02572A 26901771 10.1039/c5an02572a PMC9423764

[14] W. Alahmad, P. Varanusupakul, P. Varanusupakul, Crit. Rev. Anal. Chem. **53**, 233 (2023). 10.1080/10408347.2021.1949695 34304654 10.1080/10408347.2021.1949695

[15] T. Kaneta, W. Alahmad, P. Varanusupakul, Appl. Spectrosc. Rev. **54**, 117 (2019). 10.1080/05704928.2018.1457045

[16] World Health Organization, Burden of disease attributable to unsafe drinking-water, sanitation and hygiene, 2019 update (2023), p. 8. https://www.who.int/publications/i/item/9789240075610

[17] Y. Xia, J. Si, Z. Li, Biosens. Bioelectron. **77**, 774 (2016). 10.1016/j.bios.2015.10.032 26513284 10.1016/j.bios.2015.10.032

[18] E. Carrilho, A.W. Martinez, G.M. Whitesides, Anal. Chem. **81**, 7091 (2009). 10.1021/ac901071p 20337388 10.1021/ac901071p

[19] K. Yamada, T.G. Henares, K. Suzuki, D. Citterio, Angew. Chem. Int. Ed. **54**, 5294 (2015). 10.1002/anie.201411508 10.1002/anie.201411508 25864471

[20] E.M. Fenton, M.R. Mascarenas, G.P. López, S.S. Sibbett, A.C.S. Appl, Mater. Interfaces **1**, 124 (2009). 10.1021/am800043z 10.1021/am800043z 20355763

[21] J. Nie, Y. Liang, Y. Zhang, S. Le, D. Li, S. Zhang, Analyst **138**, 671 (2013). 10.1039/C2AN36219H 23183392 10.1039/c2an36219h

[22] S. Karita, T. Kaneta, Anal. Chem. **86**, 12108 (2014). 10.1021/ac5039384 25423320 10.1021/ac5039384

[23] S. Karita, T. Kaneta, Anal. Chim. Acta **924**, 60 (2016). 10.1016/j.aca.2016.04.019 27181645 10.1016/j.aca.2016.04.019

[24] Z. Nie, C.A. Nijhuis, J. Gong, X. Chen, A. Kumachev, A.W. Martinez, M. Narovlyansky, G.M. Whitesides, Lab Chip **10**, 477 (2010). 10.1039/B917150A 20126688 10.1039/b917150a PMC3065124

[25] S.M.Z. Hossain, J.D. Brennan, Anal. Chem. **83**, 8772 (2011). 10.1021/ac202290d 22029903 10.1021/ac202290d

[26] M.M. Mentele, J. Cunningham, K. Koehler, J. Volckens, C.S. Henry, Anal. Chem. **84**, 4474 (2012). 10.1021/ac300309c 22489881 10.1021/ac300309c

[27] D.M. Cate, W. Dungchai, J.C. Cunningham, J. Volckens, C.S. Henry, Lab Chip **13**, 2397 (2013). 10.1039/C3LC50072A 23657627 10.1039/c3lc50072a

[28] K. Ogawa, T. Kaneta, Anal. Sci. **32**, 31 (2016). 10.2116/analsci.32.31 26753702 10.2116/analsci.32.31

[29] Y. Shimada, T. Kaneta, Anal. Sci. **34**, 65 (2018). 10.2116/analsci.34.65 29321460 10.2116/analsci.34.65

[30] W. Alahmad, K. Uraisin, D. Nacapricha, T. Kaneta, Anal. Methods **8**, 5414 (2016). 10.1039/C6AY00954A

[31] A. Muhammed, A. Hussen, T. Kaneta, Anal. Bioanal. Chem. **413**, 3339 (2021). 10.1007/s00216-021-03274-y 33715041 10.1007/s00216-021-03274-y

[32] A. Muhammed, A. Hussen, M. Redi, T. Kaneta, Anal. Sci. **37**, 585 (2021). 10.2116/analsci.20P325 33041309 10.2116/analsci.20P325

[33] A. Muhammed, A. Hussen, T. Kaneta, Anal. Sci. **38**, 123 (2022). 10.2116/analsci.21P215 35287213 10.2116/analsci.21P215

[34] A. Muhammed, A. Hussen, T. Kaneta, S. Afr. J. Chem. **77**, 1 (2023). 10.17159/0379-4350/2023/v77a01

[35] A. Muhammed, A. Hussen, T. Kaneta, Anal. Sci. **40**, 709 (2024). 10.1007/s44211-023-00504-4 38316712 10.1007/s44211-023-00504-4

[36] M.I. Umeda, K. Danchana, T. Fujii, E. Hino, Y. Date, K. Aoki, T. Kaneta, Talanta Open **10**, 100347 (2024). 10.1016/j.talo.2024.100347

[37] B.M. Jayawardane, I.D. McKelvie, S.D. Kolev, Talanta **100**, 454 (2012). 10.1016/j.talanta.2012.08.021 23141364 10.1016/j.talanta.2012.08.021

[38] J.M. Racicot, T.L. Mako, A. Olivelli, M. Levine, Sensors (Basel) **20**, 2766 (2020). 10.3390/s20102766 32408677 10.3390/s20102766 PMC7294414

[39] K. Danchana, H. Namba, T. Kaneta, Submitted for publication

[40] Y. Hashimoto, T. Kaneta, Anal. Methods **11**, 179 (2019). 10.1039/C8AY02298D

[41] S. Felletti, N. Marchetti, C. De Luca, M. Catani, C. Nosengo, G. Compagnin, D. Bozza, F.A. Franchina, L. Pasti, A. Cavazzini, Trac-Trends. Anal. Chem. **176**, 117740 (2024). 10.1016/j.trac.2024.117740

[42] H. Gu, L. Hu, Y. Dong, Q. Chen, Z.J. Wei, R. Lv, J. Food Compos. Anal. **131**, 106212 (2024). 10.1016/j.jfca.2024.106212

[43] Y. Sun, H. Tang, X. Zou, G. Meng, N. Wu, Curr. Opin. Food Sci. **47**, 100910 (2022). 10.1016/j.cofs.2022.100910

[44] A. Tsiasioti, P.D. Tzanavaras, Food Chem. **443**, 138577 (2024). 10.1016/j.foodchem.2024.138577 38309023 10.1016/j.foodchem.2024.138577

[45] S. Seetasang, T. Kaneta, in *Advanced Microfluidics Based Point-of-Care Diagnostics*. ed. by R. Khan, C. Dhand, S.K. Sanghi, D.S.T. Salammal, A.P. Mishra (CRC Press, Boca Raton, 2022), pp.249–270

[46] R.W. Peeling, K.K. Holmes, D. Mabey, A. Ronald, A. Sex Transm Infect. **82**, 1 (2006). 10.1136/sti.2006.024265 17151023 10.1136/sti.2006.024265 PMC2563912

[47] L. Jin, Z. Hao, Q. Zheng, H. Chen, L. Zhu, C. Wang, X. Liu, C. Lu, Anal. Chim. Acta **1100**, 215 (2020). 10.1016/j.aca.2019.11.067 31987143 10.1016/j.aca.2019.11.067

[48] J. Zhang, Y. Li, T. Zhang, Z. Zheng, H. Jing, C. Liu, Food Chem. **439**, 138179 (2024). 10.1016/j.foodchem.2023.138179 38091789 10.1016/j.foodchem.2023.138179

[49] N. Ratnarathorn, W. Dungchai, J. Anal. Chem. **75**, 487 (2020). 10.1134/S1061934820040127

[50] S. Teepoo, S. Arsawiset, P. Chanayota, Chemosensors **7**, 44 (2019). 10.3390/chemosensors7030044

[51] K. Seebunrueng, P. Naksen, P. Jarujamrus, S. Sansuk, Y. Treekamol, N. Teshima, H. Murakami, S. Srijaranai, Food Chem. **451**, 139402 (2024). 10.1016/j.foodchem.2024.139402 38678650 10.1016/j.foodchem.2024.139402

[52] C.C. Liu, Y.N. Wang, L.M. Fu, K.L. Chen, Food Chem. **249**, 162 (2018). 10.1016/j.foodchem.2018.01.004 29407920 10.1016/j.foodchem.2018.01.004

[53] W. Li, X. Ma, Y.C. Yong, G. Liu, Z. Yang, Anal. Chim. Acta **1278**, 341614 (2023). 10.1016/j.aca.2023.341614 37709421 10.1016/j.aca.2023.341614

[54] A.Y. Flores-Ramírez, R.R. González-Estrada, M.A. Chacón-López, M. de Lourdes García-Magaña, E. Montalvo-González, A. Álvarez-López, A. Rodríguez-López, U.M. López-García, Anal. Biochem. **693**, 115600 (2024). 10.1016/j.ab.2024.115600 10.1016/j.ab.2024.115600 38964698

[55] M. Yuan, C. Li, Y. Zheng, H. Cao, T. Ye, X. Wu, L. Hao, F. Yin, J. Yu, F. Xu, Talanta **266**, 125112 (2024). 10.1016/j.talanta.2023.125112 37659229 10.1016/j.talanta.2023.125112

[56] H. Sharifi, J. Tashkhourian, B. Hemmateenejad, Anal. Chim. Acta **1126**, 114 (2020). 10.1016/j.aca.2020.06.006 32736715 10.1016/j.aca.2020.06.006

[57] M. Pan, X. Han, S. Chen, J. Yang, Y. Wang, H. Li, S. Wang, Talanta **267**, 125188 (2024). 10.1016/j.talanta.2023.125188 37716240 10.1016/j.talanta.2023.125188

[58] Y. Yang, X. Zeng, C. Fu, L. Tan, N. Yang, Y. Liu, Q. Shen, J. Wei, C. Yu, C. Lu, Anal. Chim. Acta **1272**, 341497 (2023). 10.1016/j.aca.2023.341497 37355331 10.1016/j.aca.2023.341497

[59] S. Seetasang, T. Kaneta, ACS Sens. **7**, 1194 (2022). 10.1021/acssensors.2c00300 35404587 10.1021/acssensors.2c00300

[60] K. Danchana, H. Iwasaki, K. Ochiai, H. Namba, T. Kaneta, Microchem. J. **179**, 107513 (2022). 10.1016/j.microc.2022.107513

[61] K. Danchana, H. Iwasaki, Y. Thayawutthikun, P. Saetear, T. Kaneta, ACS Omega **8**, 11213 (2023). 10.1021/acsomega.2c08138 37008150 10.1021/acsomega.2c08138 PMC10061644

[62] T. Boonpoempoon, W. Wonsawat, T. Kaneta, Sci. Rep. **9**, 12951 (2019). 10.1038/s41598-019-49393-6 31506489 10.1038/s41598-019-49393-6 PMC6736875

[63] S. Buking, Y. Suedomi, D. Nacapricha, T. Kaneta, ACS Omega **4**, 15249 (2019). 10.1021/acsomega.9b02226 31552371 10.1021/acsomega.9b02226 PMC6751694

[64] D.A. Bui, P.C. Hauser, Anal. Chim. Acta **853**, 46 (2015). 10.1016/j.aca.2014.09.044 25467449 10.1016/j.aca.2014.09.044

[65] G.B. Nair, S.J. Dhoble, Luminescence **30**, 1167 (2015). 10.1002/bio.2919 26014269 10.1002/bio.2919

[66] K.T. Lau, E. McHugh, S. Baldwin, D. Diamond, Anal. Chim. Acta **569**, 221 (2006). 10.1016/j.aca.2006.03.073

[67] Ł Tymecki, R. Koncki, Anal. Chim. Acta **639**, 73 (2009). 10.1016/j.aca.2009.03.014 19345761 10.1016/j.aca.2009.03.014

[68] P. Yeh, N. Yeh, C.H. Lee, T.J. Ding, Renew. Sust. Energ. Rev. **75**, 461 (2017). 10.1016/j.rser.2016.11.011

[69] S. Seetasang, T. Kaneta, Talanta **204**, 586 (2019). 10.1016/j.talanta.2019.06.024 31357338 10.1016/j.talanta.2019.06.024

[70] J.M. Kruse, M.G. Mellon, Anal. Chem. **25**, 446 (1953). 10.1021/ac60075a018

[71] S. Mitra, M. Basak, S. Biswas, P.S.G. Pattader, Measurement **214**, 112848 (2023). 10.1016/j.measurement.2023.112848

[72] L. Khoshmaram, M. Saadati, F. Sadeghi, Microchem. J. **152**, 104344 (2020). 10.1016/j.microc.2019.104344

[73] L.M. Fu, W.J. Ju, C.C. Liu, R.J. Yang, Y.N. Wang, Chem. Eng. J. **243**, 421 (2014). 10.1016/j.cej.2013.12.096

[74] K.M.G. de Lima, Microchem. J. **103**, 62 (2012). 10.1016/j.microc.2012.01.003

[75] E.M. Paiva, J.J.R. Rohwedder, C. Pasquini, C.F. Pereira, Microchem. J. **146**, 842 (2019). 10.1016/j.microc.2019.01.074

[76] H.V. Dantas, M.F. Barbosa, A. Pereira, M.J.C. Pontes, P.N.T. Moreira, M.C.U. Araújo, Microchem. J. **135**, 148 (2017). 10.1016/j.microc.2017.08.014

[77] S. Seetasang, T. Kaneta, Anal. Chim. Acta **1135**, 99 (2020). 10.1016/j.aca.2020.08.051 33070864 10.1016/j.aca.2020.08.051

[78] U.S. Environmental protection agency, *Recognition and Management of Pesticide Poisonings* (2013), pp. 110–117

[79] S.O. Catarino, P. Felix, P.J. Sousa, V. Pinto, M.I. Veiga, G. Minas, IEEE Trans. Biomed. Eng. **67**, 365 (2020). 10.1109/TBME.2019.2913454 31034403 10.1109/TBME.2019.2913454

[80] M.H. Sorouraddin, A. Rostami, M. Saadati, Food Chem. **127**, 308 (2011). 10.1016/j.foodchem.2010.12.124

[81] T.R. Dias, M.A.S. Brasil, M.A. Feres, B.F. Reis, Sens. Actuator B-Chem. **198**, 448 (2014). 10.1016/j.snb.2014.03.018

[82] S. Seetasang, T. Kaneta, Microchem. J. **171**, 106777 (2021). 10.1016/j.microc.2021.106777

[83] T. Ito, Y. Nakamura, J. Rural Med. **3**, 5 (2008). 10.2185/jrm.3.5

